# Prolonged serologically active clinically quiescent systemic lupus erythematosus: a longitudinal study on the role of IgG anti-dsDNA and C3

**DOI:** 10.1093/rheumatology/keag382

**Published:** 2026-07-29

**Authors:** Silvia Piunno, Augusta Ortolan, Luigi Zolio, Anisur Rahman, David A Isenberg

**Affiliations:** Department of Rheumatology, Università Cattolica del Sacro Cuore, Fondazione Policlinico Universitario Agostino Gemelli IRCSS, Rome, Italy; Department of Rheumatology, Università Cattolica del Sacro Cuore, Fondazione Policlinico Universitario Agostino Gemelli IRCSS, Rome, Italy; Department of Ageing, Rheumatology and Regenerative Medicine, University College London, London, UK; Department of Medicine, University of Melbourne, St Vincent’s Hospital, Melbourne, VIC, Australia; Department of Ageing, Rheumatology and Regenerative Medicine, University College London, London, UK; Department of Ageing, Rheumatology and Regenerative Medicine, University College London, London, UK

**Keywords:** systemic lupus erythematosus, serologically active clinically quiescent, prolonged, anti-dsDNA antibodies, IgG, C3

## Abstract

**Objectives:**

To assess whether fluctuations in immunoglobulin G anti-double stranded DNA (IgG anti-dsDNA) antibodies and C3 levels predict flares and sustained activity in patients with prolonged serologically active clinically quiescent (SACQ) systemic lupus erythematosus (SLE), and to explore additional risk factors for flare.

**Methods:**

SLE patient data from the University College London Hospital Lupus cohort were collected (2020–2025). Prolonged SACQ was defined as ≥6 months of elevated IgG anti-dsDNA and/or low C3 without clinical activity (BILAG-2004 index D/E in all domains). Associations between biomarker changes and outcomes, adjusted for main confounders, were tested longitudinally with generalized estimating equations (GEE).

**Results:**

Sixty patients (531 visits) were included. Over a mean 3.3-year follow-up, 38 patients (63.3%) experienced ≥1 flare. Baseline characteristics were comparable between patients with and without flares, except for longer follow-up duration (*P* = 0.005) and a higher prevalence of anti-Ro positivity (*P* = 0.018) among those who developed flares. In GEE-models, higher IgG anti-dsDNA and lower C3 levels independently predicted flares and sustained activity at the subsequent visit, including moderate-to-severe cases.

**Conclusion:**

Most SACQ patients developed clinical flares. Changes in IgG anti-dsDNA and C3 levels emerged as strong predictors of flare in prolonged SACQ patients in a visit-by-visit analysis, supporting close clinical monitoring and highlighting the value of serological trends despite the presence of prolonged clinical quiescence and sustained biomarker abnormalities.

Rheumatology key messages

- Most SACQ SLE patients developed clinical flares during follow-up, suggesting that this condition is rarely benign.
- Longitudinal changes in IgG anti-dsDNA and C3 strongly predicted flares even in the setting of prolonged SACQ.
- Close clinical and serologic monitoring is crucial to improve flare prevention strategies in this specific subgroup.

## Introduction

Systemic lupus erythematosus (SLE) is a potentially sinister autoimmune rheumatic disease characterized by a heterogeneous pattern of clinical and serological manifestations. Anti-double-stranded DNA (anti-dsDNA) antibodies are found in 70–80% of SLE patients and represent a highly specific diagnostic marker of the disease [1, 2]. According to several reports, anti-dsDNA antibodies correlate with disease activity and may predict disease exacerbations [3–5], particularly renal flares [6, 7], although not all anti-dsDNA antibodies share the same pathogenic potential. The immunoglobulin G (IgG) isotype more often comprises high-affinity subgroups with limited cross-reactivity, which correlate most strongly with disease activity and renal involvement [8, 9], whereas the IgM isotype may be protective and associate with quiescence [10, 11]. With less specificity than anti-dsDNA antibodies, hypocomplementemia may also be a predictor of disease flare [5, 12, 13], notably for C3, as C4 may remain chronically reduced due to genetic background [14, 15]. However, not all patients with serological activity inevitably experience flare over time. In this respect, in 1979 a subset of SLE patients with persistently elevated anti-dsDNA antibody levels and/or low complement in the absence of clinical activity was identified and designated to be serologically active clinically quiescent (SACQ) [16]. Although this condition is well recognized and accounts for 6–25% of all patients with SLE [17–20], the reliability of changes in serological biomarker levels as predictors of disease flare in this distinctive population has not been demonstrated conclusively. Against this backdrop, the primary aim of our study was to assess whether IgG anti-dsDNA antibody and C3 levels predict flares and sustained disease activity in prolonged SACQ patients. We also investigated additional potential risk factors that might contribute to flare onset.

## Methods

### Patients and definitions

Data from the University College London (UCL) Hospital Lupus Cohort was collected between November 2020 and April 2025, including demographic, clinical, laboratory and treatment information. All patients fulfilled the revised American College of Rheumatology (ACR) or the Systemic Lupus International Collaborating Clinics (SLICC) criteria for SLE [21, 22] and met the inclusion criteria for prolonged serologically active, clinically quiescent (SACQ) disease. Only patients with at least one follow-up visit were included.

Prolonged serologically active (SA) was defined as IgG anti-dsDNA antibody level >10 IU/ml or C3 <0.9 g/l or both for at least six months, including a minimum of two consecutive visits (case definition period). These values were based on the normal range for these tests in our clinical laboratory. Serological quiescence (SQ) was where neither of these conditions were met. T0 was defined as the first timepoint during follow-up when the patient entered a SACQ state. Accordingly, the ‘index date’ (i.e. the start of follow-up for flare assessment) varied across patients, depending on the individual time frame required to meet the definition of serological activity during the case definition period.

Disease activity was assessed using the British Isles Lupus Assessment group 2004 (BILAG-2004) index [23]. Clinically quiescent (CQ) was defined as grade D or E in all domains. Clinically active (CA) was identified as having a grade A, B or C in any domain.

Based on these definitions, at any following visit, a patient could be in one of four mutually exclusive states: SACQ, SACA, SQCQ and SQCA. This study focused on the patients who were in SACQ at baseline and followed their clinical and serological course thereafter.

Flares were defined as the development of a new BILAG grade A, B or C in any organ system compared with the previous visit. Sustained disease activity, defined as disease exacerbations extending over two or more consecutive timepoints (i.e. ongoing flares) was also assessed. According to severity, flares and disease activity were further categorized as mild (grade C) or moderate-to-severe (grade A or B). Patients could be taking a stable dose of antimalarials, immunosuppressants (conventional or biologics) and/or prednisolone at the maximum dose of 7.5 mg/day. Treatment de-escalation during the case definition period was permitted, whereas patients who underwent any kind of treatment escalation were excluded. Adjustments to immunosuppressive therapies involving different drugs at doses equivalent to the previous regimens for reasons unrelated to disease flare (e.g., drug intolerance, pregnancy planning) were allowed.

The study was conducted in accordance with the Declaration of Helsinki. All data were obtained from routine clinical practice, and no patients were subjected to additional questionnaires or research-specific procedures. No individualized or identifiable data are presented in this study. Therefore, ethical approval and informed consent were not required.

### Laboratory measures

IgG anti-dsDNA antibodies (normal <10 IU/ml) were measured by enzyme-linked immunosorbent essay (ELISA). The C3 fraction of complement levels was analysed using laser nephelometry, with a normal reference range of 0.9–1.8 g/l. Laboratory tests were performed on the same day as the clinical visit.

### Statistical analysis

The baseline characteristics between SACQ patients with and without flares were compared using the χ^2^ test or Mann–Whitney test, as appropriate. As part of the descriptive analysis, continuous variables were presented as mean ± standard deviation (SD) or as median (interquartile range, IQR), as appropriate.

Associations between the independent variables (IgG anti-dsDNA and C3) and disease activity outcomes over time were tested using generalized estimating equations (GEE). This method is well suited for longitudinal data, as it incorporates all available timepoints, handles missing data robustly and accounts for intra-subject correlation. Specifically, the models were designed to evaluate four distinct outcomes:

- clinical flare, defined as the new occurrence of BILAG A, B or C grade in at least one domain;
- sustained clinical disease activity, defined as the presence of BILAG A, B or C grade in at least one domain over two or more consecutive episodes;
- moderate-to-severe clinical flare, defined as the new occurrence of BILAG A or B grade in at least one domain; and
- sustained moderate-to-severe clinical disease activity, defined as the presence of BILAG A or B grade in at least one domain over two or more consecutive episodes.

The independent variables and covariates (female sex, age, therapy tapering) in the GEE models were treated as ‘lagged’ to maintain temporal accuracy, with each predictor measured at the visit prior to the corresponding outcome, thereby minimizing the potential for reverse causality. For easier interpretation, IgG anti-dsDNA antibody levels were divided by 10 to represent the risk of outcome for a 10-unit increase in IgG anti-dsDNA antibody titre, whereas for C3, the risk was assessed per 0.1-unit decrease. Results are presented as odds ratios (OR) with corresponding 95% confidence intervals (95% CI), indicating the likelihood of change in the outcome. As part of the GEE framework, a working correlation structure must be specified. An exchangeable structure was selected, as it was verified to be the most appropriate in the present dataset. A *P*-value <0.05 was considered statistically significant.

In the GEE analysis, all available values of the independent variables were included, covering the full range of measurements without truncation and thereby preserving the variability of each predictor while minimizing potential distortion. For this reason, a restricted cubic spline analysis was performed for C3 to model potential non-linear effects.

Due to the observational setting, a sensitivity analysis was performed adjusting the GEE model for time between visits.

All statistical analysis were performed using Stata/SE 17.0 (StataCorp, College Station, TX, USA).

## Results

A total of 60 patients (52 females, 86.7%) fulfilled the SACQ criteria as described, with 45 (75%) patients with elevated IgG anti-dsDNA antibodies (i.e. >10 IU/ml), 22 (36.7%) with low C3 (i.e. <0.9 g/l) and 7 (11.7%) with both these during the case definition period. The study population had a mean age of 47 (SD 13) years. The cohort contributed a total of 531 visits, with a mean of 10.5 visits (SD 4.0), occurring at a mean interval of 123 days (SD 73). The mean follow-up was 3.33 ± 0.93 years. Among all patients receiving low-dose steroid therapy during the case definition period, only two were taking doses of prednisolone 7 mg/day and 7.5 mg/day, respectively, while the remaining patients were receiving doses not exceeding 5 mg/day. Three patients died from non-lupus-related causes during follow-up, with data included up to the last available visit, each contributing nine, seven and six timepoints.

**Figure 1:**
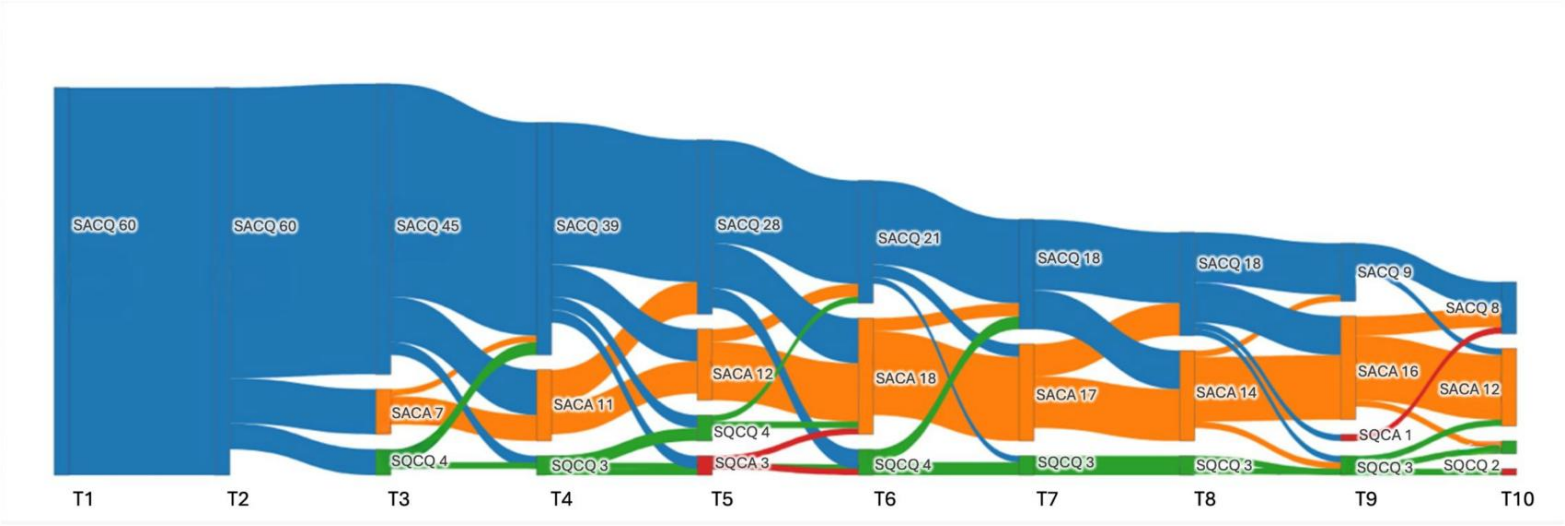
Alluvial diagram illustrating the dynamic transitions of 60 SACQ patients across clinical and serological states during follow-up. Each column represents a timepoint (T0–T10) and flow width reflects the number of patients. Colours indicate different clinical–serological states: SACQ (blue), SACA (orange), SQCQ (green), SQCA (red). SACQ, serologically active clinically quiescent; SACA, serologically active clinically active; SQCQ, serologically active clinically quiescent; SQCA, serologically quiescent clinically active.

Thirty-eight patients (63.3%) had at least one flare over the observation period. Of these, 36 (60%) patients shifted from SACQ to SACA status in at least one visit, whereas the numbers of patients with at least one transition from SACQ to SCQC and SQCA were 15 (25%) and three (5%), respectively. The dynamic transitions of the cohort among the various timepoints are illustrated in Fig. 1.

A total of 117 flares were observed, including 31 moderate-to-severe events in 15 patients. The most common flares were haematological, recorded in 25 (41.7%) patients, followed by musculoskeletal, mucocutaneous and renal flares, observed in 20 (33.3%), 15 (25%) and eight (13.3%) patients, respectively. Two patients experienced cardiorespiratory flares, whereas only one patient had a constitutional flare. Overall, 14 patients developed multi-domain flares, involving at least two domains simultaneously. The distribution of flares by domain and severity is reported in Supplementary Tables S1 and S2, respectively, while Supplementary Table S3 shows the distribution of patients according to the number of flares experienced during follow-up. Mean time to first flare was 524 days (SD 239 days), with a median of 476 (IQR 311–735). Mean time to first moderate-to-severe flare was 554 days (SD 272 days), with a median of 504 (IQR 308–735).

Most baseline characteristics (gender, age, ethnicity, comorbidity, disease duration, type of disease involvement at diagnosis, and therapies) were comparable between patients who experienced at least one flare and those who remained clinically quiescent over time. Notably, patients who developed flares had a longer follow-up duration (*P* = 0.005) and a higher prevalence of anti-Ro positivity (*P* = 0.018), as reported in Table 1.

**Table 1:** Baseline characteristics of SACQ patients with or without flare.

**Variable,** n **(%)**	**No flare (**n** = 22)**	**≥1 flare (**n** = 38)**	*P*-value
Female sex	18 (82)	35 (92)	0.232
Disease duration (years)	18.9 (12.1)	17.0 (11.6)	0.452
Follow-up (years)	2.3 (1.2)	3.2 (1.1)	**0.005**
Age (years)	44.9 (14.0)	46.0 (12.8)	0.695
Ethnicity			0.319
Caucasian	16 (76)	18 (47)	
Afro-Caribbean	2 (10)	3 (8)	
South Asian	1 (5)	5 (13)	
East Asian	1 (5)	4 (10)	
Other	1 (5)	7 (18)	
Comorbidities			
Comorbidity (RDCI ≥1)	16 (73)	24 (63)	0.449
RDCI (value)	1.5 (1.4)	1.0 (1.3)	0.206
Involved domains at onset			
Constitutional	4 (18)	7 (18)	0.982
Mucocutaneous	20 (64.5)	11 (35.5)	0.844
Neuropsychiatric	3 (14)	1 (3)	0.100
Musculoskeletal	15 (68)	28 (74)	0.649
Cardiorespiratory	2 (9)	7 (18)	0.329
Renal	5 (23)	7 (18)	0.688
Haematological	4 (18)	7 (18)	0.982
Antibody profile^a^			
Anti-dsDNA	19 (86)	36 (95)	0.343
Anti-Sm	3 (14)	10 (26)	0.237
Anti-RNP	6 (27)	13 (34)	0.377
Anti-Ro	5 (23)	22 (58)	**0.018**
Anti-La	1 (5)	5 (13)	0.248
ANCA	2 (9)	3 (8)	0.508
RF	1 (5)	1 (3)	0.379
LAC	2 (9)	5 (13)	0.382
aCL	6 (27)	16 (42)	0.246
aβ2GPI	7 (32)	10 (26)	0.354
Therapy			
Pred^b^	9 (41%)	22 (58%)	0.205
HCQ	14 (64%)	26 (68%)	0.705
MMF	4 (18%)	9 (24%)	0.618
AZA	3 (14%)	3 (8%)	0.475
MTX	0 (0%)	2 (5%)	0.274
TAC	2 (9%)	1 (3%)	0.269
CsA	0 (0%)	0 (0%)	N/A
BEL	1 (5%)	0 (0%)	0.185

Significant results are reported in bold. RDCI: Rheumatic Disease Comorbidity Index; anti-dsDNA: anti-double-stranded DNA; anti-Sm: anti-Smith; anti-RNP: anti-ribonucleoprotein; ANCA: antineutrophil cytoplasmic antibody; RF: rheumatoid factor; LAC: lupus anticoagulant; aCL: anticardiolipin; aβ2GPI: anti–β2 glycoprotein I; Pred: prednisolone; HCQ: hydroxychloroquine; MMF: mycophenolate mofetil; AZA: azathioprine; MTX: methotrexate; TAC: tacrolimus; CsA: ciclosporin A; BEL: belimumab; N/A: not applicable (no CsA users in the case definition period).

a Antibody positivity ever during the disease course.

b Prednisolone ≤7.5 mg/day.

**Figure 2:**
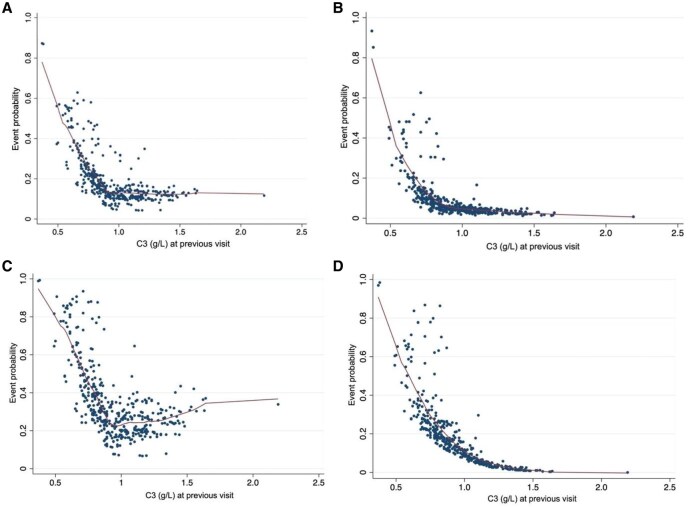
Restricted cubic spline models illustrating the non-linear associations between complement C3 levels at the previous visit and the predicted probability of subsequent clinical events, corresponding to (**A**) any flare, (**B**) BILAG A–B flare, (**C**) any disease activity, (**D**) BILAG A–B disease activity. Blue points represent individual observations; red curves show spline-based predictions of the events.

In GEE models, both IgG anti-dsDNA antibody and C3 levels at the preceding visit emerged as independent predictors of subsequent disease activity. Specifically, a 10-unit increase in IgG anti-dsDNA antibodies was associated with a higher likelihood of flares (OR 1.04, 95% CI 1.01–1.08) and sustained disease activity (OR 1.08, 95% CI 1.04–1.13). Similarly, a 0.1-unit decrease in C3 levels was independently associated with a higher risk of both flares (OR 1.26, 95% CI 1.11–1.43) and sustained activity (OR 1.23, 95% CI 1.12–1.37). The same was true for moderate-to-severe flares (OR 1.07, 95% CI 1.03–1.12; OR 1.37, 95% 1.11–1.69) as well as moderate-to-severe activity (OR 1.08, 95% CI 1.04–1.13; OR 1.59, 95% CI 1.33–1.89). This association remained robust in the model adjusted for therapeutic de-escalations over follow-up. Results from the GEE models are reported in Tables 2 and 3. Additionally, the sensitivity analysis, adjusted for time between visits, confirmed the consistent association of IgG anti-dsDNA and C3 with the outcomes (Supplementary Tables S4 and S5). Of note, time between visits was relatively homogeneous (IQR ∼3–6 months), and visit intervals were generally consistent across patients, reflecting routine clinical follow-up in a specialized lupus clinic (Supplementary Tables S6 and S7). Cubic spline analysis for C3 is shown in Fig. 2. Across all panels corresponding to the four outcomes (any flare, BILAG A–B flare, any disease activity and BILAG A–B disease activity), a sharp increase in outcome risk was observed at lower C3 concentrations (i.e. <0.9 g/l), with the association flattening at higher values.

**Table 2:** Multivariable GEE models evaluating incident flares or persistent disease activity as outcomes.

	Outcome							
	Flare (Model 1)		Flare (Model 2)		Disease activity (Model 1)		Disease activity (Model 2)	
Variables	OR (95% CI)	*P*-value	OR (95% CI)	*P*-value	OR (95% CI)	*P*-value	OR (95% CI)	*P*-value
IgG anti-dsDNA (+10 UI/mL)	**1.04** **(1.01–1.08)**	**0.021**	**1.04** **(1.01–1.08)**	**0.020**	**1.08** **(1.04–1.13)**	**<0.0001**	**1.08** **(1.04–1.13)**	**<0.0001**
C3 (−0.1 g/L)	**1.26** **(1.11–1.43)**	**<0.0001**	**1.25** **(1.11–1.43)**	**<0.0001**	**1.23** **(1.12–1.37)**	**<0.0001**	**1.23** **(1.11–1.35)**	**<0.0001**
Female sex	2.15 (0.86–5.36)	0.100	2.16 (0.86–5.43)	0.102	**2.45** **(1.18–5.10)**	**0.016**	**2.36** **(1.12–4.94)**	**0.023**
Age (years)	1.01 (0.99–1.03)	0.458	1.01 (0.99–1.03)	0.437	1.01 (1.00–1.03)	0.100	1.02 (1.00–1.03)	0.087
Pred reduction/stop	—	—	0.93 (0.39–2.25)	0.880	—	—	1.63 (0.79–3.38)	0.189
HCQ reduction/stop	—	—	2.89 (0.89–9.39)	0.078	—	—	1.30 (0.41–4.18)	0.656
IS drugs^a^ reduction/stop	—	—	1.12 (0.41–3.05)	0.830	—	—	1.05 (0.42–2.66)	0.915

Variables include the independent variables (IgG anti-dsDNA and C3) and covariates. Variables were assessed at the visit preceding the outcome (lagged). Model 1 includes sex and age as confounders. Model 2 is additionally adjusted for therapies. Significant results are reported in bold. IgG anti-dsDNA: immunoglobulin G anti-double stranded DNA antibodies; Pred: prednisolone; HCQ: hydroxychloroquine; IS drugs: immunosuppressive drugs.

a Immunosuppressive drugs include mycophenolate mofetil, methotrexate, azathioprine, tacrolimus, ciclosporin A, belimumab.

**Table 3:** Multivariable GEE models evaluating incident moderate-to-severe (BILAG A–B) flares and persistent disease activity as outcomes.

	Outcome							
	BILAG A–B flare (Model 1)		BILAG A–B flare (Model 2)		BILAG A–B disease activity (Model 1)		BILAG A–B disease activity (Model 2)	
Variables	OR (95% CI)	*P*-value	OR (95% CI)	*P*-value	OR (95% CI)	*P*-value	OR (95% CI)	*P*-value
IgG anti-dsDNA (+10 UI/mL)	**1.07** **(1.03–1.12)**	**<0.0001**	**1.07** **(1.03–1.11)**	**0.001**	**1.08** **(1.04–1.13)**	**<0.0001**	**1.08** **(1.04–1.13)**	**<0.0001**
C3 (−0.1 g/L)	**1.37** **(1.11–1.69)**	**0.004**	**1.39** **(1.12–1.72)**	**0.003**	**1.59** **(1.33–1.89)**	**<0.0001**	**1.59** **(1.31–1.89)**	**<0.0001**
Female sex	Omitted	—	Omitted	—	Omitted	—	Omitted	—
Age (years)	0.98 (0.94–1.02)	0.343	0.98 (0.94–1.01)	0.314	0.99 (0.96–1.02)	0.455	0.99 (0.96–1.02)	0.499
Pred reduction/stop	—	—	0.57 (0.13–2.36)	0.436	—	—	1.35 (0.55–3.31)	0.511
HCQ reduction/stop	—	—	Omitted	—	—	—	Omitted	—
IS drugs^a^ reduction/stop	—	—	0.80 (0.16–4.00)	0.789	—	—	1.56 (0.45–5.44)	0.486

Variables include the independent variables (IgG anti-dsDNA and C3) and covariates. Variables were assessed at the visit preceding the outcome (lagged). Model 1 includes sex and age as confounders. Model 2 is additionally adjusted for therapies. Significant results are reported in bold. ‘Omitted’ variables were excluded from the GEE model due to perfect prediction of the outcome or insufficient variability for parameter estimation. IgG anti-dsDNA: immunoglobulin G anti-double stranded DNA antibodies; Pred: prednisolone; HCQ: hydroxychloroquine; IS drugs: immunosuppressive drugs.

a Immunosuppressive drugs include mycophenolate mofetil, methotrexate, azathioprine, tacrolimus, ciclosporin A, belimumab.

## Discussion

In this study, we comprehensively characterized a longitudinal cohort of prolonged SACQ SLE patients, identifying serological predictors of clinical reactivation. Over time, >60% of the cohort experienced disease flares, and both increased IgG anti-dsDNA and low C3 levels at the preceding visit independently predicted activity, highlighting the dynamic nature of SACQ disease and the prognostic value of these traditional biomarkers also in this subgroup.

Few studies have investigated the fluctuations of anti-dsDNA antibodies and complement in relation to disease activity in prolonged SACQ subjects, and a precise correlation with flares has not been conclusively established. In a cohort of 56 SACQ patients, Steiman *et al.* [17] found no correlation between increases in anti-dsDNA antibodies or complement fluctuations and subsequent clinical flares, emphasizing the need for alternative predictive biomarkers. In a subsequent study, the same authors found no association between disease activity and fluctuations in anti-dsDNA and antichromatin antibody isotypes and IgG subclasses in 23 SACQ patients [24], examined under the hypothesis that clinical quiescence might reflect a predominance of less pathogenic isotypes. Finally, in a cohort of 21 SACQ individuals, Ng *et al.* observed that patients with anti-dsDNA antibody levels at baseline exceeding five times the upper limit of normal experienced a shorter time to flare, although no direct association between the antibody profile and disease activity onset was investigated in a longitudinal design [18].

Since late 2020, the UCL Hospital routine laboratory transitioned from an assay detecting both IgG and IgM anti-dsDNA antibodies to one selective for the IgG isotype, given its stronger pathogenic role and correlation with disease activity [8–10], in contrast to IgM, which promotes antigen clearance and modulates autoreactive responses [9, 11]. Complement analysis was restricted to C3 in our study, as C4 may remain persistently low in SLE patients irrespective of disease activity, largely due to the strong influence of C4 gene copy number on serum levels [25, 26]. C4 levels were not routinely monitored in all patients as part of standard clinical practice and were therefore unavailable at several timepoints.

A notable finding of our study was the high proportion of prolonged SACQ subjects developing clinical activity during follow-up: over 60% of the cohort transitioned to SACA at least once. With the exception of the study by Steiman *et al.* [17], which reported a flare incidence comparable to ours (58.9%) over nearly four decades of follow-up—likely inflating the cumulative incidence—the rate observed in our work was higher than those reported in other studies [19, 20] (34.3% and 32.9%, respectively). This discrepancy may be attributable to differences in the definition and follow-up of SACQ patients, which in the other studies were based on the SLE Disease Activity Index-2000 (SLEDAI-2K) [27]. By contrast, we applied the more sensitive BILAG-2004 index [23], providing a detailed evaluation across nine organ systems and better capturing the onset of clinical activity [28–30]. In fact, Ng *et al.*, who also used the BILAG-2004 index, reported a flare incidence of 81% over 5 years. This finding, along with persistent disease activity in a small subset of refractory patients, may explain the continued detection of SACA across multiple timepoints in several individuals. Additionally, the use of the BILAG-2004 index leads to a precise identification of patients in true clinical remission, excluding those with minimal manifestations potentially lupus-related (e.g. arthralgia or mild haematological changes), who would be classified as SACQ under SLEDAI-2K. Of note, Ng *et al.* used a broader SACQ definition than ours, including also subjects with BILAG score <6 (i.e. with mild disease activity including BILAG C).

The presence of additional risk factors for clinical activity was investigated in our cohort, and no significant differences emerged between flare and non-flare groups with respect to baseline demographic or clinical features, including sex, age, ethnicity, comorbidities, disease duration, presenting symptoms and therapies (Table 1). Interestingly, anti-Ro positivity was more frequent in patients who developed flares. Another study suggests that the association between anti-SSA antibodies and flare risk may be context-dependent, emerging only within specific SACQ subgroups [19]. Our finding supports a potential association, although confirmation in larger prolonged SACQ cohorts is needed. A significant discrepancy between the two categories was also observed in the duration of follow-up, with flare patients having longer follow-up periods. Accordingly, this finding should be viewed in this context, as differences in follow-up duration may reflect differential opportunities for flare detection rather than intrinsic predictors of disease activity. This observation is consistent with the longitudinal pattern of clinical and serological activity among our SACQ cohort, illustrated in Fig. 1.

Other studies investigating the presence of risk factors for flare among persistent SACQ patients have yielded heterogeneous results. Huang *et al.* found no distinguishing baseline features in their study of 170 SACQ [20], whereas the multicentre study by Ding *et al.* of 990 SACQ subjects identified a subgroup at highest risk of severe flare and organ damage, characterized by elderly men with major organ involvement at diagnosis [19]. Collectively, these findings suggest that our cohort may not have been sufficiently large to identify a precise flare-prone phenotype, but also that traditional demographic or clinical factors may be less informative than dynamic serological markers in identifying patients at risk of reactivation.

In fact, in GEE models, higher IgG anti-dsDNA antibody and/or lower C3 levels at the preceding visit independently predicted disease activity at the subsequent timepoint, even after the adjustment for potential confounders, thereby confirming their genuine predictive value while appropriately accounting for repeated measures.

More recent studies have revisited the prognostic role of serological activity within treat-to-target frameworks, including definition of remission in SLE (DORIS) [31] and lupus low disease activity state (LLDAS) [32]. A systematic review reported a heterogeneous and overall modest association between abnormal serology and subsequent flare risk, highlighting methodological variability and limitations across studies [33]. Notably, most of the included studies were conducted in non-uniform SLE populations, comprising patients with active disease, clinically stable but not in remission, or SACQ status assessed at a single time point. Similarly, an integrated analysis of belimumab trials showed that the combination of clinical remission and normal serological status was associated with a lower risk of flares. However, these findings derive from populations with active disease at baseline and rely on categorical definitions of serology, whereas our study evaluates dynamic changes in biomarker levels in relation to flare risk within a prolonged SACQ population [34].

Compared with our approach, previous observational studies focusing on prolonged SACQ employed different statistical strategies, centred on arbitrarily selected timepoints before flare and therefore overlooking the full longitudinal trajectory [17], including cohorts with only a few patients providing paired SACQ-flare samples [24], or estimating flare onset on the basis of a single anti-dsDNA antibody measurement obtained during the SACQ period [18]. Key methodological differences across studies are summarized in Table 4.

**Table 4:** Comparison of studies evaluating the association between anti-dsDNA antibodies/complement levels and disease activity in patients with prolonged SACQ.

Study	Variable	Patients and timepoints	Disease activity index	Statistical approach	Predictive of disease activity
Our study	IgG anti-dsDNA, C3	60 patients, 531 timepoints	BILAG-2004 index	GEE longitudinal models	Yes
Steiman *et al*. [17]	anti-dsDNA, C3/C4	56 patients, timepoints not reported (∼336–392 estimated)	SLEDAI-2K	Pre-flare *vs* persistent SACQ/SQCQ^a^	No
Steiman *et al.* [24]	Isotypes (IgM, IgA, IgG) and IgG subclasses (IgG1–4) of anti-dsDNA and anti-chromatin antibodies	23 patients, timepoints not reported (38 samples; only 5 patients with paired SACQ + flare samples)	SLEDAI-2K	Mostly qualitative, limited GEE	No
Ng *et al.* [18]	anti-dsDNA, anti-NCS	21 patients, baseline measurement only	BILAG-2004 index^b^	Time-to-event analysis, Spearman’s correlation	No; patients with anti-dsDNA > 5× ULN or anti-NCS positivity at baseline: earlier flares (not longitudinal)

a Biomarker levels at the two visits preceding flare (flare group) were compared with those measured at the third-last and second-last visits in patients who remained SACQ or became SQCQ at their last visit (non-flare group).

b BILAG-2004 score <6 as inclusion criteria. SACQ: serologically active clinically quiescent; IgG anti-dsDNA: immunoglobulin G anti-double-stranded DNA antibodies; anti-NCS: anti-nucleosome antibodies; BILAG-2004: British Isles Lupus Assessment group 2004; SLEDAI-2K: SLEDAI 2000; GEE: generalized estimating equations; ULN: upper limit of normal.

Limitations of our study include its observational design, the modest length of follow-up and the temporal definition of SACQ, which required persistent serological abnormalities for ≥6 months, as in most other studies [18–20]. In fact, adopting a longer time threshold to define SACQ could better capture prolonged disease quiescence and improve specificity. However, our study has several strengths. Firstly, it incorporated frequent and numerous timepoints, maximizing the predictive significance of biomarker fluctuations. Secondly, the longitudinal model, evaluating each variable at the preceding time point in relation to disease activity at the subsequent one, enabled a comprehensive assessment of biomarker dynamics rather than a static comparison of variable changes. Finally, use of the BILAG-2004 index enabled finer discrimination of SACQ status and more sensitive detection of clinical activity.

In conclusion, prolonged SACQ patients represent a distinct subgroup of SLE in whom persistent serological activity without clinical manifestations makes flare prediction challenging. To our knowledge, this study provides the first longitudinal evidence of a direct correlation between rising IgG anti-dsDNA titres and/or declining C3 levels and subsequent disease activity in this population, supporting their genuine predictive value and providing important insight into its temporal dynamics. The high likelihood of developing clinical flares indicates that the SACQ state is significant, and regular monitoring of IgG anti-dsDNA antibodies and C3 levels may support a more personalized and timely approach to disease management, helping to reduce flare-related morbidity and long-term organ damage.

## Supplementary Material

keag382_Supplementary_Data

## Data Availability

Data are available from the corresponding author upon reasonable request.
