# Supplementary material for: Prolonged serologically active clinically quiescent systemic lupus erythematosus: a longitudinal study on the role of IgG anti-dsDNA and C3
Source: Rheumatology (Oxford). 2026 Jul 29;65(8):keag382. doi: 10.1093/rheumatology/keag382 (PMC13441167; doi:10.1093/rheumatology/keag382)
Supplement: Supplementary file 1 [file keag382_supplementary_data.docx]

# Supplementary Materials

**Table S1**. Flare distribution by domain

| **Domain** | **Flares, n** | **Flare patients, n** |
| --- | --- | --- |
| Constitutional | 1 | 1 |
| Mucocutaneous | 23 | 15 |
| Neuropsychiatric | 0 | 0 |
| Musculoskeletal | 28 | 20 |
| Cardiorespiratory | 3 | 2 |
| Gastrointestinal | 0 | 0 |
| Ophthalmic | 0 | 0 |
| Renal | 15 | 8 |
| Haematological | 48 | 25 |

**Table S2.** Flare distribution by severity and corresponding number of affected patients

| Flare severity | Flares, n | Flare patients, n (%)* |
| --- | --- | --- |
| BILAG A index | 6 | 5 (8.3) |
| BILAG B index | 25 | 15 (25) |
| BILAG C index | 86 | 35 (58.3) |
| Total | 117 | 38 (63.3) |

*Patients with at least one flare of the corresponding grade. Note: all patients with grade A flares also had grade B flares. BILAG, British Isles Lupus Assessment Group.

**Table S3**. Frequency of patients with any number of flares over follow-up

| **Total flares, n** | **Patients, n (%)** |
| --- | --- |
| **0** | 22 (36.7) |
| **1** | 15 (25) |
| **2** | 12 (20) |
| **3** | 4 (6.7) |
| **4** | 2 (3.3) |
| **5** | 1 (1.7) |
| **6** | 3 (5) |
| **8** | 1 (1.7) |
| **Total** | 60 (100) |

## **Table S4.** Multivariable GEE models evaluating incident flares or persistent disease activity as outcomes

| **Outcome** | | | | | | | | |
| --- | --- | --- | --- | --- | --- | --- | --- | --- |
|  | **Flare (Model 1)** |  | **Flare (Model 2)** |  | **Disease activity (Model 1)** | | **Disease activity (Model 2)** | |
| **Variables** | OR (95% CI) | p-value | OR (95% CI) | p-value | OR (95% CI) | p-value | OR (95% CI) | p-value |
| **IgG anti-dsDNA (+10 UI/mL)** | **1.04**  **(1.00 – 1.08)** | **0.023** | **1.04**  **(1.00 – 1.08)** | **0.023** | **1.07**  **(1.02 – 1.12)** | **0.002** | **1.07**  **(1.03 – 1.11)** | **0.002** |
| **C3 (-0.1 g/L)** | **1.03**  **(1.01 – 1.04)** | **<0.0001** | **1.02**  **(1.01 – 1.04)** | **0.001** | **1.02**  **(1.01 – 1.03)** | **<0.0001** | **1.02**  **(1.01 – 1.03)** | **0.001** |
| **Female sex** | 2.15  (0.86 – 5.38) | 0.100 | 2.16  (0.85 – 5.44) | 0.104 | **2.16**  **(1.02 – 4.56)** | **0.043** | 2.06  (0.97 – 4.39) | 0.059 |
| **Age (years)** | 1.01  (0.99 – 1.03) | 0.465 | 1.01  (0.99 – 1.03) | 0.438 | **1.02**  **(1.00 – 1.04)** | **0.026** | 1.02  (1.00 – 1.04) | 0.024 |
| **Pred reduction/stop** | – | – | 0.93  (0.39 – 2.25) | 0.880 | – | – | 1.54  (0.73 – 3.26) | 0.253 |
| **HCQ reduction/stop** | – | – | 2.89  (0.89 – 9.41) | 0.078 | – | – | 1.44  (0.44 – 4.73) | 0.550 |
| **IS drugs* reduction/stop** | – | – | 1.11  (0.41 – 3.05) | 0.831 | – | – | 0.95  (0.37 – 2.44) | 0.920 |
| **Time between visits (months)** | 1.00  (0.88 – 1.15) | 0.965 | 0.99  (0.87 – 1.14) | 0.981 | **0.77**  **(0.56 – 0.88)** | **<0.0001** | **0.77**  **(0.67 – 0.89)** | **0.001** |

Variables include the independent variables (IgG anti-dsDNA and C3) and covariates. Variables were assessed at the visit preceding the outcome (lagged). Model 1 includes sex, age and time between visits as confounders. Model 2 is additionally adjusted for therapies. Significant results are reported in bold. IgG anti-dsDNA, Immunoglobulin G anti-double stranded DNA antibodies; Pred, prednisolone; HCQ, hydroxychloroquine; IS drugs, immunosuppressive drugs.

*Immunosuppressive drugs include mycophenolate mofetil, methotrexate**,** azathioprine, tacrolimus, cyclosporin A, belimumab.

## **Table S5**. Multivariable GEE models evaluating incident moderate-to-severe (BILAG A–B) flares and persistent disease activity as outcomes

|  |  |  |  | **Outcome** |  |  |  |  |
| --- | --- | --- | --- | --- | --- | --- | --- | --- |
|  | **BILAG A–B flare (Model 1)** | | **BILAG A–B flare (Model 2)** | | **BILAG A–B**  **disease activity**  **(Model 1)** | | **BILAG A–B**  **disease activity**  **(Model 2)** | |
| **Variables** | OR (95% CI) | p-value | OR (95% CI) | p-value | OR (95% CI) | p-value | OR (95% CI) | p-value |
| **IgG anti-dsDNA (+10 UI/mL)** | **1.07**  **(1.03 – 1.12)** | **0.001** | **1.07**  **(1.03 – 1.12)** | **0.001** | **1.07**  **(1.03 – 1.13)** | **0.001** | **1.07**  **(1.02 – 1.11)** | **0.002** |
| **C3 (-0.1 g/L)** | **1.03**  **(1.01 – 1.06)** | **0.004** | **1.03**  **(1.01 – 1.06)** | **0.003** | **1.04**  **(1.02 – 1.06)** | **<0.0001** | **1.04**  **(1.02 – 1.06)** | **<0.0001** |
| **Female sex** | Omitted | – | Omitted | – | Omitted | – | Omitted | – |
| **Age (years)** | 0.98  (0.94 – 1.02) | 0.350 | 0.98  (0.94 – 1.02) | 0.320 | 0.99  (0.97 – 1.02) | 0.762 | 0.99  (0.97 – 1.02) | 0.800 |
| **Pred reduction/stop** | – | – | 0.57  (0.14 – 2.36) | 0.436 | – | – | 1.25  (0.50 – 3.14) | 0.637 |
| **HCQ**  **reduction/stop** | – | – | Omitted | – | – | – | Omitted | – |
| **IS drugs* reduction/stop** | – | – | 0.80  (0.16 – 4.00) | 0.788 | – | – | 1.35  (0.38 – 4.81) | 0.639 |
| **Time between visits (months)** | 0.99  (0.80 – 1.23) | 0.943 | 0.99  (0.79 – 1.24) | 0.959 | **0.68**  **(0.54 – 0.84)** | **<0.0001** | **0.68**  **(0.54 – 0.84)** | **0.001** |

Variables include the independent variables (IgG anti-dsDNA and C3) and covariates. Variables were assessed at the visit preceding the outcome (lagged). Model 1 includes sex, age and time between visits as confounders. Model 2 is additionally adjusted for therapies. Significant results are reported in bold. “Omitted” variables were excluded from the GEE model due to perfect prediction of the outcome or insufficient variability for parameter estimation. IgG anti-dsDNA, Immunoglobulin G anti-double stranded DNA antibodies; Pred, prednisolone; HCQ, hydroxychloroquine; IS drugs, immunosuppressive drugs.

*Immunosuppressive drugs include mycophenolate mofetil, methotrexate**,** azathioprine, tacrolimus, cyclosporin A, belimumab.

**Table S6**. Summary statistics for time intervals between visits (months)

| **Visit** | **Mean (months)** | **SD** | **p25** | **p50** | **p75** |
| --- | --- | --- | --- | --- | --- |
| 1 | 0.00 | 0.00 | 0.00 | 0.00 | 0.00 |
| 2 | 4.79 | 1.80 | 3.68 | 4.37 | 5.95 |
| 3 | 4.69 | 1.64 | 3.79 | 4.37 | 5.98 |
| 4 | 5.30 | 2.92 | 3.84 | 4.40 | 6.21 |
| 5 | 5.13 | 2.54 | 3.37 | 4.48 | 6.21 |
| 6 | 4.49 | 1.86 | 3.22 | 4.14 | 5.98 |
| 7 | 4.70 | 1.53 | 3.91 | 4.37 | 5.98 |
| 8 | 4.38 | 1.84 | 3.10 | 4.14 | 6.18 |
| 9 | 4.28 | 1.90 | 2.99 | 3.91 | 5.98 |
| 10 | 4.75 | 1.80 | 3.42 | 4.37 | 5.98 |
| 11 | 3.59 | 1.28 | 2.99 | 3.35 | 4.14 |
| 12 | 4.07 | 1.26 | 3.45 | 4.14 | 4.14 |
| 13 | 3.80 | 1.35 | 2.91 | 3.71 | 4.68 |
| 14 | 2.19 | 0.39 | 1.84 | 2.07 | 2.50 |
| 15 | 2.74 | 1.07 | 2.12 | 3.19 | 3.37 |
| 16 | 2.63 | 0.52 | 2.30 | 2.73 | 2.96 |
| 17 | 2.59 | 1.51 | 1.35 | 2.68 | 3.83 |
| 18 | 2.38 | 0.12 | 2.30 | 2.38 | 2.46 |
| 19 | 4.21 | — | 4.21 | 4.21 | 4.21 |
| 20 | 1.77 | — | 1.77 | 1.77 | 1.77 |
| **Total** | **4.07** | **2.41** | **2.79** | **4.14** | **5.98** |

Visit refers to the sequential visit number within the follow-up period. Values are presented as mean, standard deviation (SD), and percentiles (p25, p50 [median], p75).

**Table S7**. Summary statistics for cumulative follow-up time across visits (months)

| **Visit** | **Mean**  **(months)** | **SD** | **p25** | **p50** | **p75** |
| --- | --- | --- | --- | --- | --- |
| 1 | 0.00 | 0.00 | 0.00 | 0.00 | 0.00 |
| 2 | 4.79 | 1.80 | 3.68 | 4.37 | 5.95 |
| 3 | 9.40 | 2.40 | 7.82 | 9.08 | 10.58 |
| 4 | 14.41 | 3.85 | 11.50 | 13.86 | 16.33 |
| 5 | 19.30 | 5.25 | 15.08 | 20.01 | 21.84 |
| 6 | 23.17 | 5.72 | 18.86 | 23.69 | 26.68 |
| 7 | 27.60 | 6.25 | 23.23 | 27.84 | 31.28 |
| 8 | 31.39 | 6.96 | 25.76 | 32.31 | 36.24 |
| 9 | 34.79 | 7.14 | 29.70 | 36.15 | 38.64 |
| 10 | 38.89 | 7.50 | 34.05 | 38.99 | 42.78 |
| 11 | 39.40 | 6.53 | 36.86 | 40.25 | 43.46 |
| 12 | 41.42 | 7.17 | 41.16 | 42.78 | 45.54 |
| 13 | 42.23 | 7.69 | 39.66 | 43.46 | 47.93 |
| 14 | 41.77 | 8.44 | 39.10 | 44.12 | 45.77 |
| 15 | 42.18 | 7.78 | 36.14 | 43.73 | 48.21 |
| 16 | 44.80 | 7.65 | 38.89 | 46.70 | 50.74 |
| 17 | 47.41 | 6.53 | 42.70 | 49.56 | 52.10 |
| 18 | 45.08 | 6.18 | 40.73 | 45.08 | 49.44 |
| 19 | 44.91 | — | 44.91 | 44.91 | 44.91 |
| 20 | 46.69 | — | 46.69 | 46.69 | 46.69 |
| **Total** | **20.19** | **14.61** | **7.82** | **17.71** | **32.12** |

Visit refers to the sequential visit number within the follow-up period. Values are presented as mean, standard deviation (SD), and percentiles (p25, p50 [median], p75).
